# Temporal and regional variations in use, equity and quality of antenatal care in Egypt: a repeat cross-sectional analysis using Demographic and Health Surveys

**DOI:** 10.1186/s12884-019-2409-1

**Published:** 2019-07-26

**Authors:** Miguel Pugliese-Garcia, Emma Radovich, Nevine Hassanein, Oona M. R. Campbell, Karima Khalil, Lenka Benova

**Affiliations:** 1 Faculty of Public Health and Policy, London School of Hygiene and Tropical Medicine, Tavistock Place, London, WC1H 9SH UK; 2 Faculty of Epidemiology and Population Health, London School of Hygiene and Tropical Medicine, Keppel Street, London, WC1E 7HT UK; 3 Gynuity Health Projects, Egypt team, 220 East 42nd, New York, NY 10017 USA; 4 Public Health Consultant, New Delhi, India; 5 Department of Public Health, Institute of Tropical Medicine, Antwerp, Belgium

**Keywords:** Antenatal care, Egypt, care quality, Equity, Demographic and Health survey, health-seeking, maternal health, effective coverage

## Abstract

**Background:**

Egypt has seen substantial decreases in maternal mortality and reached near universal coverage for antenatal care (ANC). The objective of this paper is to describe the changes over time (1991–2014) in the use of ANC in Egypt, focusing on sector of provision (public versus private), and the content and equity of this care, to inform future policies for improving maternal and newborn health.

**Methods:**

We used Demographic and Health surveys (DHS) conducted in Egypt in 1995, 2000, 2005, 2008 and 2014 to explore national and regional trends in ANC. To assess content of care, we calculated the percentage of ANC users who reported receiving seven ANC components measured in DHS in 2014.

**Results:**

During the period under consideration, the percentage of women in need of ANC who received facility-based ANC increased from 42 to 90%, the majority of which was private-sector ANC. The mean number of ANC visits among ANC users increased over time from 7.5 (95% confidence interval [CI] = 7.1–7.9) in 1991–1995 to 9.7 (95%CI 9.6–9.9) in 2010–2014. In 2010–2014, 44% of women using public ANC reported eight or more visits compared to 71% in private ANC. In the same period, 24% of ANC users received all seven care components. This percentage ranged from 10% of women reporting fewer than four ANC visits to 29% of women reporting eight or more. The poorest ANC users received all seven measured components of care less often than the wealthiest (20% versus 28%, *p*-value< 0.001).

**Conclusions:**

Egypt’s improvements in ANC coverage were characterized by decreasing reliance on public services and a rising number of ANC visits. However, despite rising ANC coverage, less than a third of women received the seven essential ANC components measured at least once during pregnancy, with differences between poorer and wealthier women. Policymakers need to ensure that high ANC coverage translates into equity-focused interventions targeting ANC quality. Further research needs to support this effort by assessing the determinants behind poor quality of ANC and evaluating potential interventions.

**Electronic supplementary material:**

The online version of this article (10.1186/s12884-019-2409-1) contains supplementary material, which is available to authorized users.

## Background

Access to good quality antenatal and skilled delivery care can prevent the majority of maternal and perinatal deaths [1–5]. Specifically, the role of antenatal care (ANC) in monitoring pregnancy is three-fold: prevention of conditions that may have unfavourable effects on the health of the mother and child, treatment of health problems, and provision of information about pregnancy, childbirth and the postnatal period to the mother [6–8]. The World Health Organization (WHO) released new recommendations on routine ANC for pregnant women in November 2016 [9]. In addition to increasing the recommended number of ANC contacts from a minimum of four to eight, this recommendation highlighted the importance of quality of care received by women during pregnancy. Following this recommendation, global attention has been shifting from predominantly measuring utilisation (coverage) of ANC to additional considerations of equity and quality of this care in order to tackle remaining preventable morbidity and mortality among women and their babies [10–13].

In the last three decades, Egypt has seen substantial improvements in maternal survival: a decrease in maternal mortality from 174 per 100,000 live births in 1992–3 [14] to a level of around 33–42 per 100,000 live births by 2015 [15, 16]. This progress was achieved through various strategies to increase utilisation of care, such as provider training, introduction of standards of care, facility upgrades and public awareness campaigns. Some of these interventions were specifically targeted to maternal services, and some were wider programmes aimed at the health system as a whole. Female education levels and modern contraceptive prevalence rate, both of which are linked to better reproductive and maternal health outcomes, also increased during this time period [17]. Yet, substandard care by obstetricians, absent or poor quality antenatal care, and delays in recognising problems and seeking care were identified as the most important avoidable causes of maternal death [17]. ANC is particularly important as a vehicle for communicating information about complications of pregnancy and delivery, and discussing birth preparedness in general.

Egypt has reached near universal coverage with 1+ ANC visits (> 90% in the 2010–2014 period) [18]. With high levels of care utilisation, it is imperative that the focus turns to identifying and closing remaining differences in the provision of good quality care (including both under- and over-medicalisation) [12, 19] and in equitable access to care [20]. Egypt is also a regionally and globally important middle-income country (e.g. in terms of economy, population, culture, and exporting doctors) with a growing population and pressures on public healthcare, evidenced by a growing proportion of care provided by the private sector [18, 21, 22]. Thus, Egypt’s health system and socioeconomic and political context provide an excellent opportunity to formulate targeted policy interventions to ensure equity and quality of care to further improve maternal and newborn health and inform other countries with similar circumstances.

The objective of this paper is to describe the changes over time in the use of ANC in Egypt, focusing on the sector of provision (public versus private), and the content and equity of ANC, nationally and by region, in order to inform future policies.

## Methods

### Data

We used the five most recent Demographic and Health surveys (DHS) conducted in Egypt in 1995, 2000, 2005, 2008 and 2014. DHS are cross-sectional nationally representative household surveys with a multistage cluster sampling strategy. Their model questionnaires are adapted to each country’s circumstances and include questions on household and individual characteristics, fertility and family planning, maternal and child health and details on ANC and delivery care. The DHS receive government permission and follow ethical practices including informed consent and assurance of confidentiality [18].

### Population

All ever-married women aged 15–49 with a live birth in the surveys’ five-year recall period were included in the analysis. In results, we refer to the surveys using the duration of their recall periods (e.g., 1995 survey is 1991–1995). We examined women’s self-report of ANC source, number of visits, and components of care for the pregnancy leading to the most recent live birth.

### Indicators and definitions

We analysed DHS surveys covering the period from 1991 to 2014 to calculate the percentage of women using facility-based ANC and private facility-based ANC at the national and regional levels. We also calculated the distribution of the number of visits reported by users of facility-based ANC, by survey. Using the most recent DHS from 2014, we estimated the distribution of ANC visits among women in need of ANC, by wealth quintile. We calculated the percentage of women using ANC in the public and private sector who received a minimum of four visits (as recommended by WHO guidelines during the period covered by the surveys) and eight visits (as recommended since 2016). We also estimated the percentage of women receiving various components of ANC, by wealth quintile and region, from the most recent survey. Finally, we assessed the percentage of women receiving all components of care reported on this survey, stratified by number of visits to ANC, sector of service, household wealth and type of residence.

### Women in need of ANC and using ANC

Women were considered to be in need of ANC if they had a live birth in the survey’s five-year recall period. Women were considered ANC users if they reported receiving ANC in a health facility at least once during pregnancy. Women who did not recall how many visits they had (521 women, 1.7% of the combined sample of women from all five surveys) or who reported over 30 visits (73 women, 0.17%) were classified as ANC users when measuring the number of women using ANC, but excluded from our calculations of numbers of ANC visits and components of care.

### Sector of ANC provision (public or private)

Women were categorised according to the sector of provision (public or private) they reported using. Public-sector providers included public, government or social security health facilities. Private-sector providers included five types: private hospitals, private clinics, private doctors, NGOs, and other private providers. We chose to categorize the few women accessing both public and private providers for ANC as private sector as the DHS does not ask how many visits were at each type of provider nor what services they obtained at each. These women constitute a very small proportion of ANC users (807 women, 2.8%), and we felt that their experience of care was probably more similar to users of only private sector ANC than to users of only public sector ANC. Across the five surveys, there were 64 women (< 0.05%) who reported receiving ANC only at home or in “other non-medical” locations. Since these women were not considered to be users of ANC (defined through facility use), they were excluded from the analysis of sector of provision and components of care.

### Household wealth

Equity in ANC use was examined using wealth quintiles from the most recent DHS survey covering the period 2010–2014. Quintiles were created measuring households’ asset ownership and creating appropriate thresholds [23, 24]. To avoid the use of subpopulations with very small samples (*n* < 100) in our analysis of content of care, we combined quintiles one and two to represent women from the poorest 40% of households, and quintiles 4 and 5 to represent the wealthiest 40%.

### Subnational regions

All five DHS used the same six major administrative regions to produce subnational estimates. These include Urban Governorates (four cities without rural populations: Cairo, Alexandria, Port Said, Suez), urban Lower Egypt (ULE), rural Lower Egypt (RLE), urban Upper Egypt (UUE), rural Upper Egypt (RUE), and Frontier Governorates (Red Sea, New Valley, Matrouh, North Sinai and South Sinai Governorates). North and South Sinai were excluded from the 2014 survey due to security issues. While national estimates were not affected by this exclusion due to a small percentage of the total population (< 1%) residing there, caution is needed when comparing 2010–2014 Frontier Governorates estimates to estimates from previous surveys.

### Components of care

In the most recent survey (2014), women using ANC were asked whether they received seven specific care components during their pregnancy: having been weighed, having had a blood sample taken, having had a urine sample taken, receiving information on signs of possible complications during pregnancy, receiving or buying iron tablets or syrup, having had their blood pressure measured, and receiving tetanus shots. With the exception of tetanus, women were asked if they received each component at least once during pregnancy. They were not asked how many times, during which visits, from which provider or location, or at what gestational age. Tetanus protection was estimated using an algorithm used previously, and considered vaccination received during the index pregnancy and in previous pregnancies [12]. Women responding, “don’t know”, or having a missing value for any component, were classified as not receiving the component.

### Analysis

Data analysis was conducted in Stata SEv14 (College Station, TX), using the *svyset* command to account for survey design of each survey (sample weights, clustering and stratification). Tabulations and Chi-square tests were used to provide descriptive statistics and to assess the statistical significance of different distributions. To capture the extent of variability in the number of ANC visits reported, we report ranges, means and medians across the different surveys and wealth quintiles. Details of the sample on each survey is provided in Additional file 1.

## Results

### National trends in overall and private ANC use 1991–2014

**Fig. 1:**
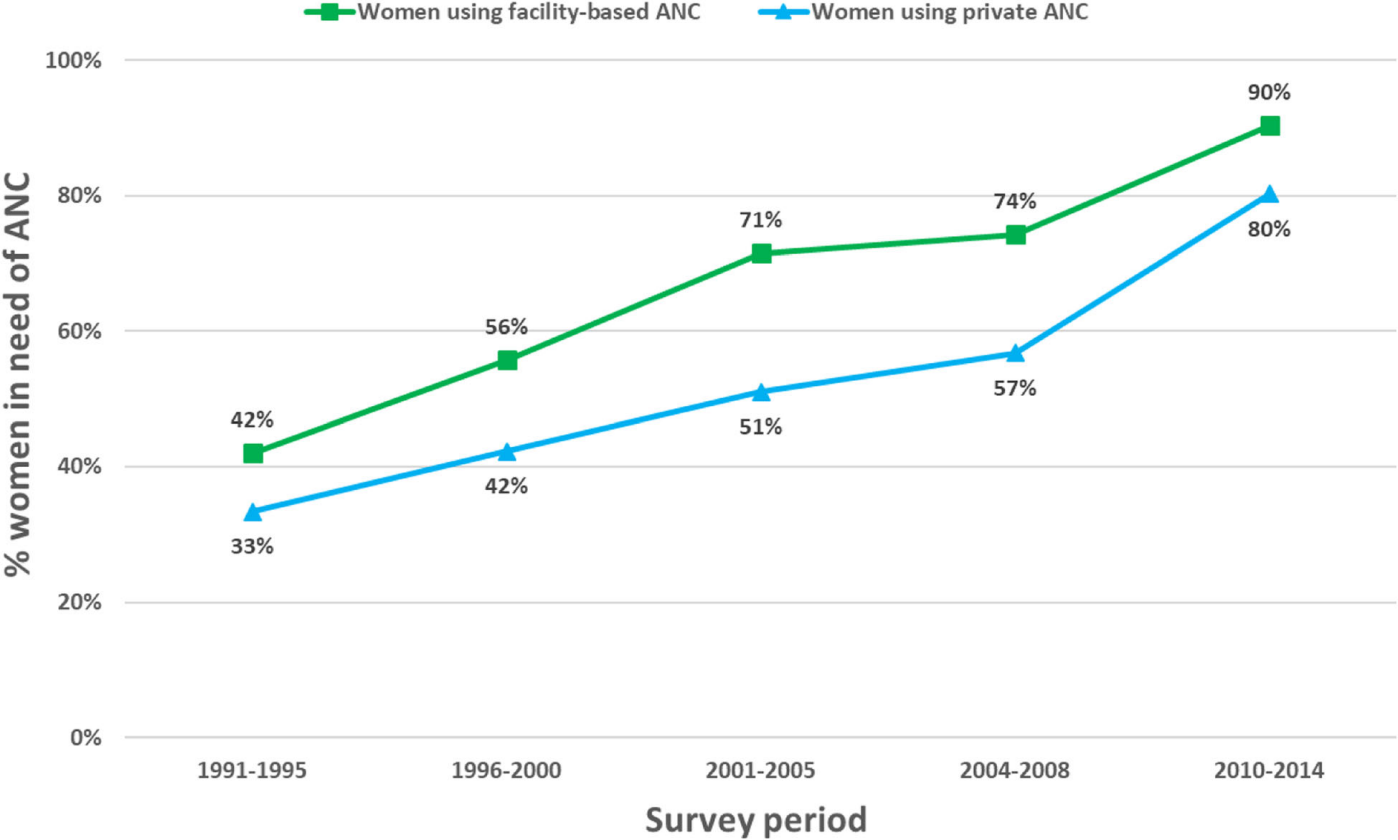
Percentage of facility-based ANC users by sector of provision and survey period

Between 1991–1995 and 2010–2014, the recall periods for the earliest and the most recent survey, the percentage of women in need of ANC who reported receiving facility-based ANC increased from 42 to 90% (Fig. 1). Similarly, the percentage of women in need of ANC who received private ANC rose from 33 to 80%. The proportion of all facility-based ANC users obtaining care from the private sector (i.e., the private sector market share), declined slightly from 78% in 1991–1995 to 71% in the period 2000–2005, and then increased to 89% in 2010–2014 period.

**Fig. 2:**
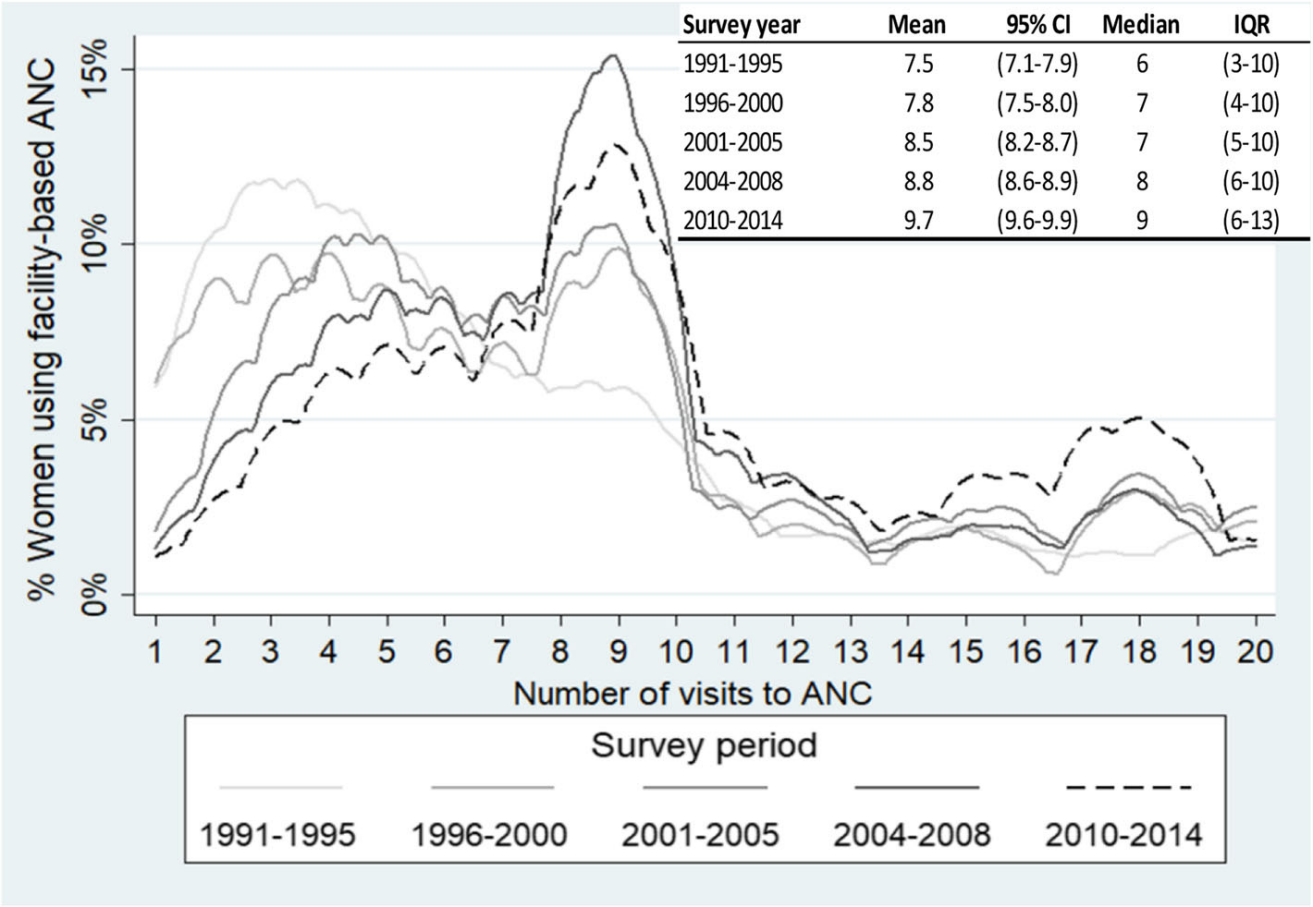
Distribution of visits reported by users of facility-based ANC, by survey

The mean number of ANC visits during pregnancy among ANC users increased over time (Fig. 2) from 7.5 (95% confidence interval [CI] = 7.1–7.9) in 1991–1995 to 9.7 (95%CI 9.6–9.9) in 2010–2014. The median number of visits increased from 6 (interquartile range [IQR] 3–10) in 1991–1995 to 9 (IQR 6–13) in 2010–2014.

### Regional trends in ANC use and private ANC use between 1991–1995 and 2010–2014

**Fig. 3:**
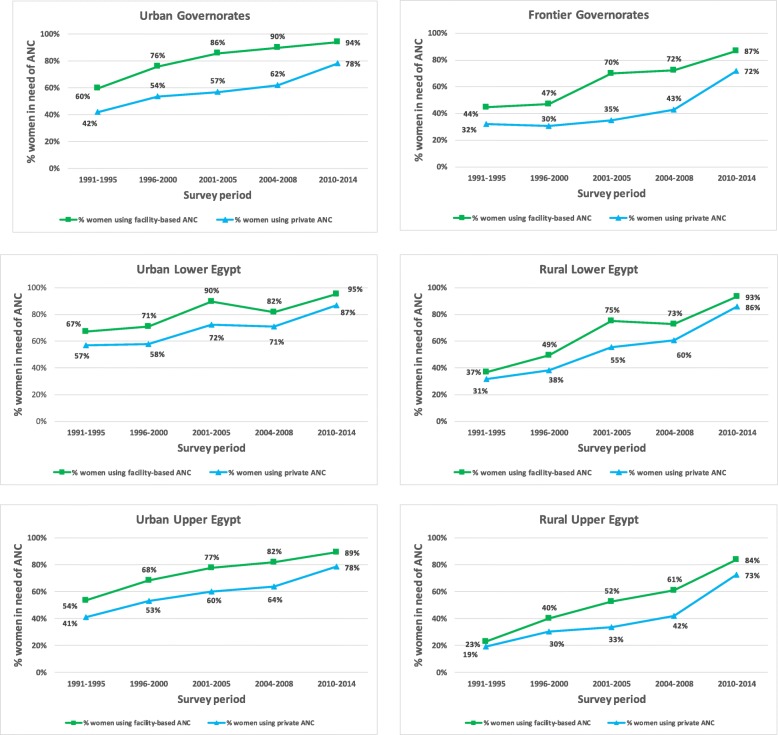
Percentage of facility-based ANC users by sector of provision and survey period in each region

The use of ANC increased in all six regions over the time period assessed (Fig. 3). In the 1991–1995 period, the percentage of women in need who used ANC ranged from 23% in RUE to 67% ULE. These two regions were also on the extremes of the range in 2010–2014: 84% in RUE and 95% in ULE. The percentage of women in need of ANC who used private-sector providers also increased in the same period, with RUE and ULE registering the lowest (19%) and highest (56%) use of private services in 1991–1995 among women in need of ANC. In the 2010–2014 period, both RUE and Frontier Governorates had the lowest percentage of women in need of ANC using private services—72%—while ULE had the highest—87%. There was a reduction in regional differences in overall ANC use over the time period: from 44 to 12 percentage points (pp) of difference from 1991 to 1995 to 2010–2014 between the regions with highest and lowest overall ANC. There was a reduction in differences across regions in private sector ANC, although not as large as for overall coverage, going from 38 pp. to 14 pp. Across the six regions, RUE registered both the largest absolute and the largest relative increase in ANC use (61 pp. and 267%, respectively), followed by RLE (56 pp. and 153%, respectively).

### Equity of ANC use in the 2010–2014 period

**Fig. 4:**
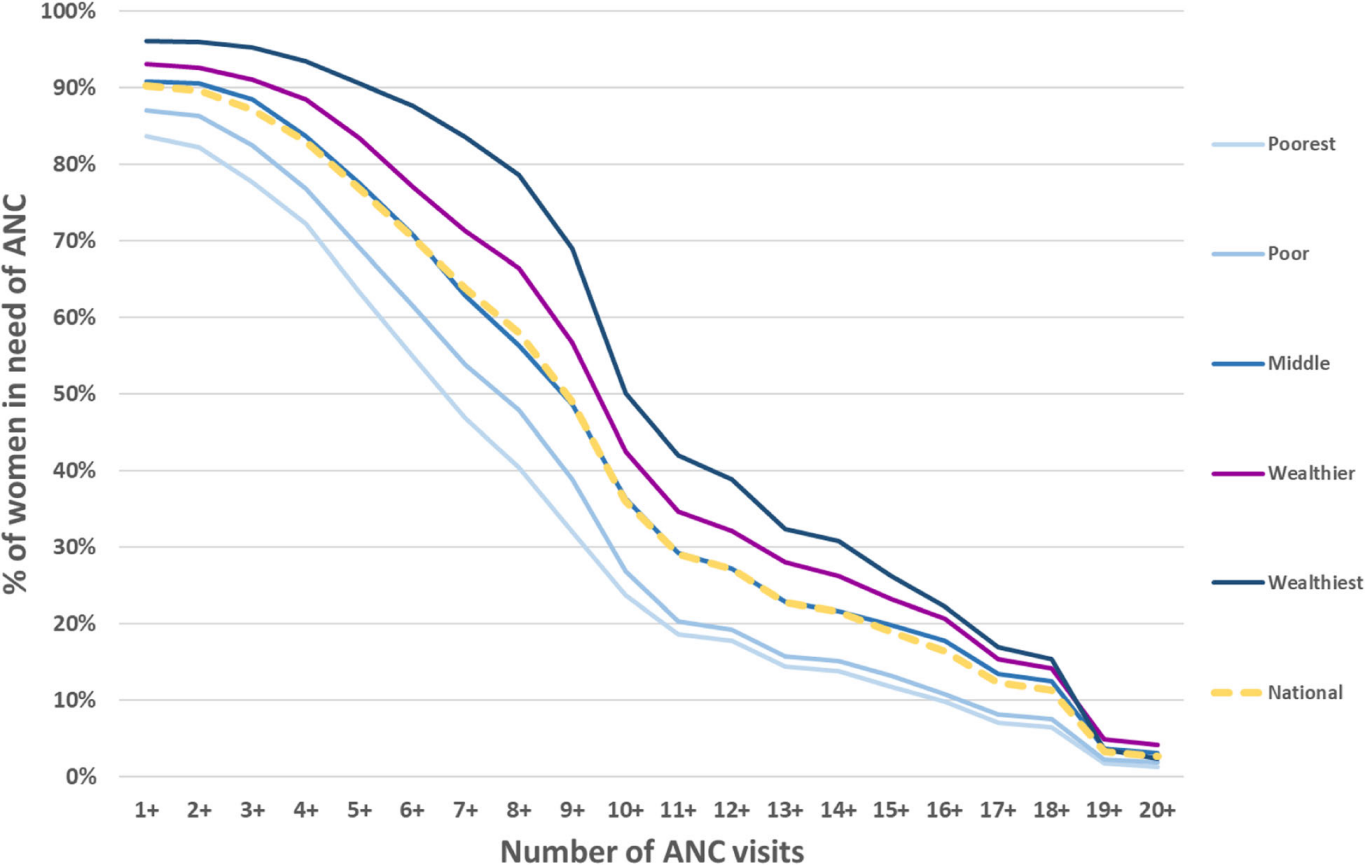
Distribution of the number of antenatal care visits among women in need of ANC, by wealth quintile in the period 2010–2014

The cumulative distribution of numbers of ANC visits differed between wealth quintiles (Fig. 4). The percentage of women reporting four or more visits ranged from 72% in the poorest quintile to 93% in the wealthiest. More than half of the women in every wealth quintile reported six or more visits, and over 40% reported eight or more.

**Fig. 5:**
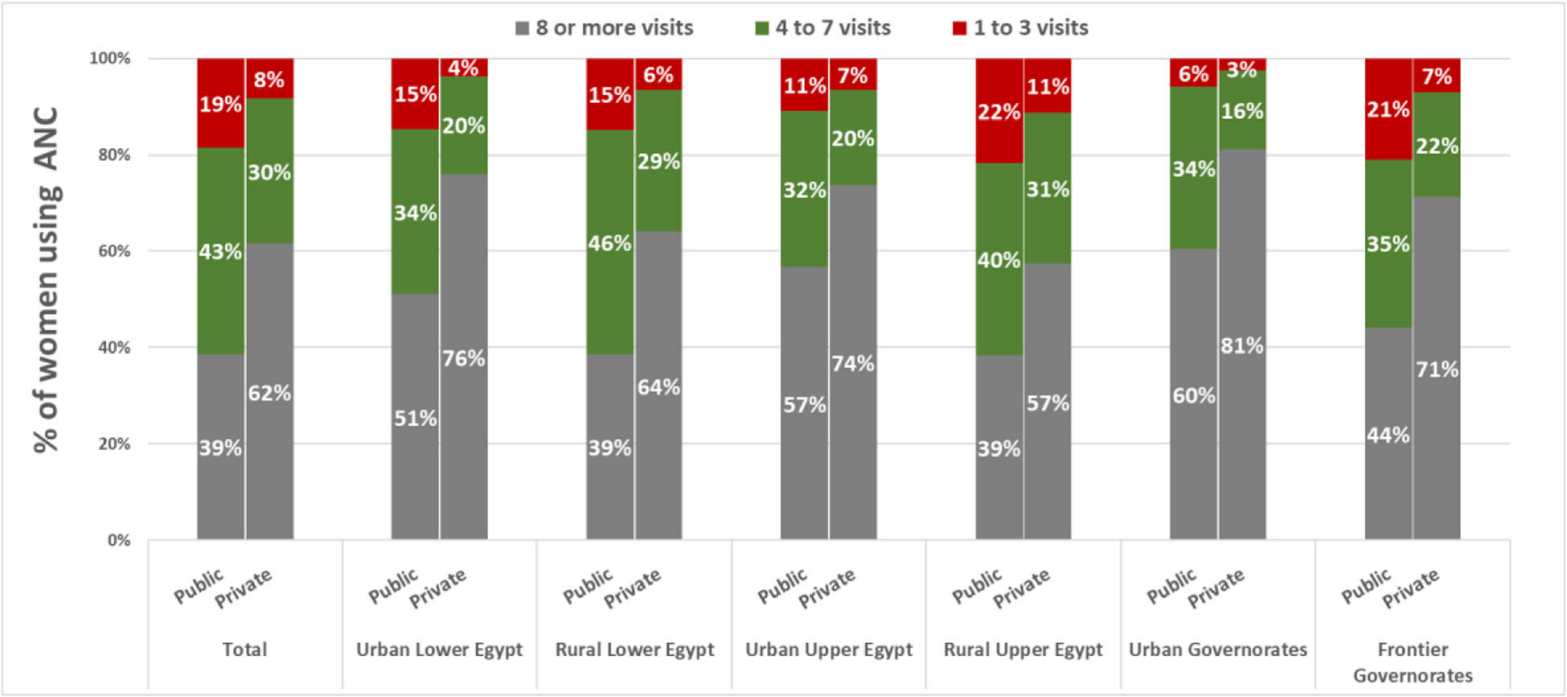
Percentage of women using ANC with one to three, four to seven, and eight or more ANC visits, by region and sector of provision in the 2010 and 2014

After stratifying by sector and region (Fig. 5), there were substantial differences in the distribution of visits across the sectors in 2010–2014. For example, 44% of women using public ANC reported eight or more visits compared to 71% in the private sector, whereas 21% of public and 7% of private-sector users reported one to four visits. Urban Governorates had the largest percentage of women reporting eight or more visits in the public (60%) and private (81%) sectors. Frontier Governorates had the highest percentage of women with fewer than four visits within the public sector (22%) and RUE in the private sector (11%).

### Components of care on the period 2010–2014

#### Subnational differences in components of ANC care

**Fig. 6:**
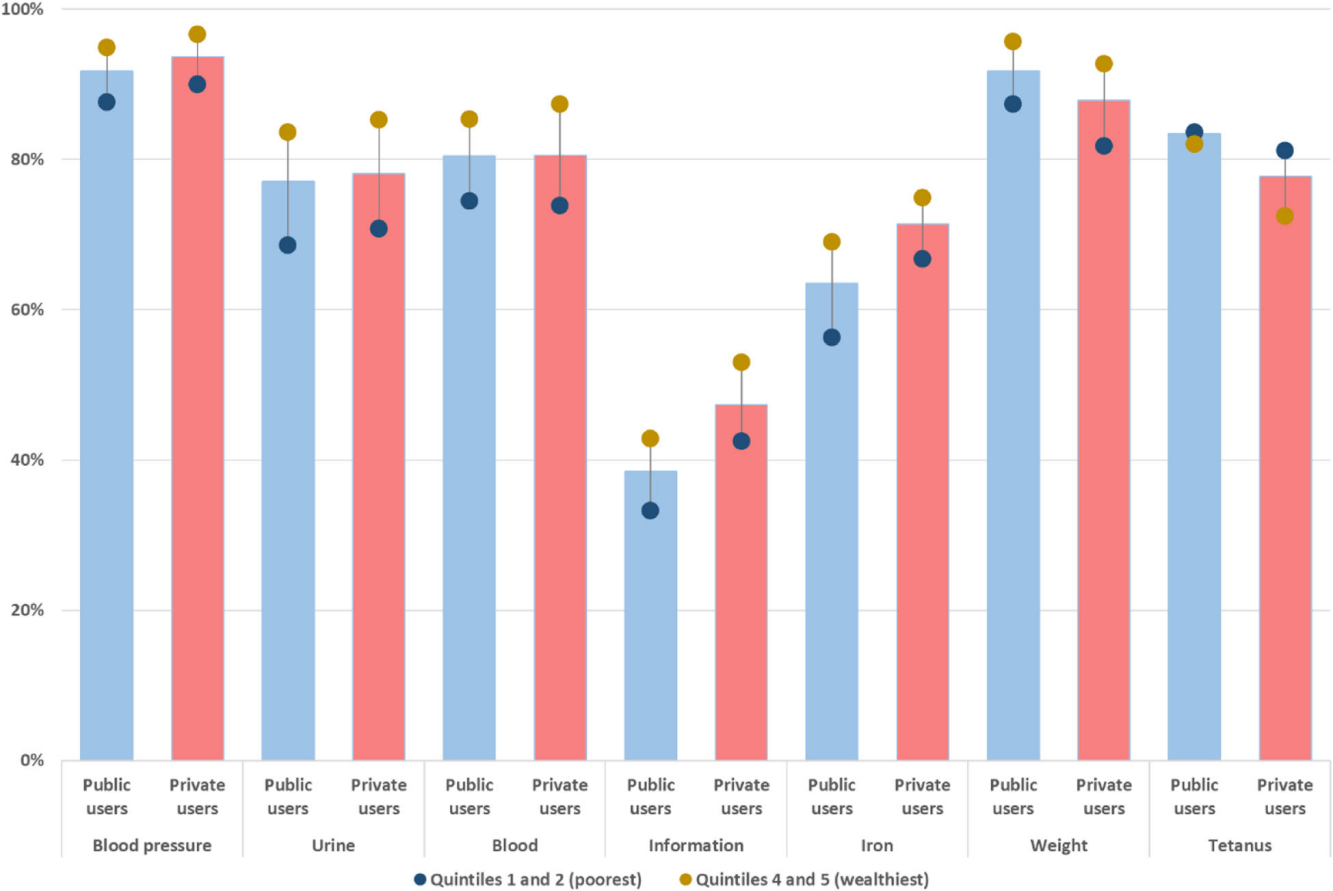
Percentage of women using public and private ANC that received each of the seven components care studied, stratified by wealth in the period 2010–2014

From the seven ANC components collected on the 2014 survey, the provision of information on danger signs during pregnancy was the least commonly received ANC component, reported by 39% of women using public and 47% using private providers (Fig. 6). This was followed by iron supplements, reported by 64 and 71% of the women using public and private providers, respectively. In both public and private sectors, the poorest 40% of women reported receiving components less often than the wealthiest 40% of women. Tetanus vaccinations were the only component where this was not observed; in fact, the gradient was reversed and favoured the poorest among women using private-sector ANC.

**Fig. 7:**
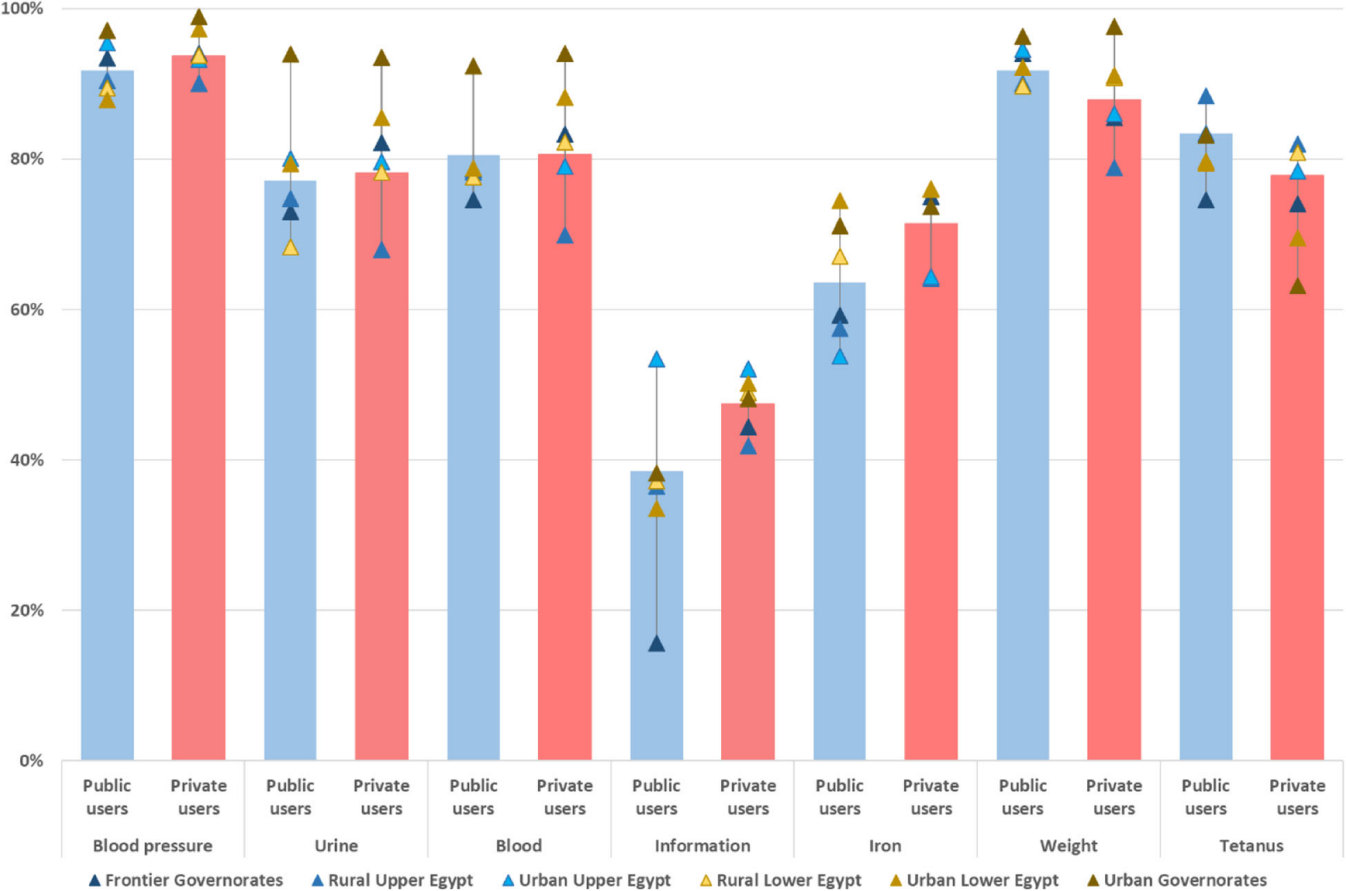
Percentage of women using public and private ANC that received each of the seven components care studied, stratified by region in the period 2010–2014

The percentage of women who received each ANC component varied by region and sector of care (Fig. 7). Large differences in the percentage of women reporting a component of care in the public sector were observed in the provision of information on danger signs, with 38 pp. between UUE and FG, and collecting urine samples, with 26 pp. between UG and RLE. In the private sector, large differences was observed in collection of urine samples, 26 pp. between UG and RUE, and weight measurements, 19 pp. between UG and RUE. The wealth and regional gradient privileging women from Urban Governorates seemed reversed for tetanus protection.

#### Receipt of all components of ANC care

Because the distribution pattern for receipt of tetanus protection differed compared to the other ANC components, we present two summaries of care content: the first includes all seven components and the second includes six (excluding tetanus protection). In the period 2010–2014, 24% of ANC users received all seven components; ranging from 10% of women reporting fewer than four ANC visits, to 29% of women reporting eight or more (Table 1). After removing the tetanus vaccinations component from our analysis, 29% of ANC users received all six ANC components, from 10% in women with fewer than four visits, to 35% in those with eight or more.

**Table 1:** Percentage of facility-based ANC users reporting receiving components of care in the period 2010–2014, by number of ANC visits and sector of provision

All seven measured ANC components										
		Sector of service			Household wealth			Residence		
Number of visits	Public	Private	*p*-value	Poorest	Wealthiest	*p*-value	Rural	Urban	*p*-value	Total
1–3	8%	10%	0.505	6%	17%	< 0.001	9%	12%	0.323	**10%**
4–7	15%	18%	0.302	16%	20%	0.071	18%	15%	0.147	**18%**
8+	27%	29%	0.439	26%	31%	0.004	29%	28%	0.389	**29%**
All	20%	25%	0.007	20%	28%	< 0.001	24%	24%	0.862	**24%**
Six ANC components (excluding tetanus protection)										
1–3	9%	11%	0.634	7%	18%	< 0.001	10%	13%	0.243	**10%**
4–7	17%	21%	0.138	18%	25%	0.004	20%	20%	0.956	**20%**
8+	31%	36%	0.090	29%	40%	< 0.001	34%	38%	0.028	**35%**
All	22%	30%	< 0.001	22%	36%	< 0.001	27%	33%	< 0.001	**29%**

*P*-values measure, for each category of antenatal care visits, the differences between sector, wealth quintile and residence type respectively

Comparing the public and private sectors, 25% of women using private ANC received all seven components versus 20% of women using public (*p*-value< 0.007). After removing tetanus vaccinations, these values increased to 30% for private ANC and 22% for public (*p*-value< 0.001). However, differences between sectors were not significant after stratifying ANC users by whether they reported one to three visits, four to seven, or eight or more.

The poorest 40% of ANC users received all seven measured components of care less often than the wealthiest 40% (20%, compared to 28%, *p*-value< 0.001). After stratifying by the three categories representing the number of ANC visits, this difference remained statistically significant among women reporting one to three visits, (6 and 17%, *p*-value< 0.001) and eight or more visits (26 and 31%, *p*-value = 0.004). After dropping tetanus vaccinations, which were more common among poorer women, 36% of the women in the wealthiest group reported all six components, compared to 22% in the poorest (p-value< 0.001). Differences between both groups remained statistically significant in women reporting one to three visits (7% of the poorest and 18%, of the wealthiest, p-value< 0.001), four to seven (18 and 25%, *p*-value< 0.004) and eight and more (29 and 40%, *p*-value< 0.001).

Differences between women from rural and urban areas were not significant in the analysis of seven ANC components. In the analysis of six components, differences were observed by women’s residence: 27% of rural women reported receiving all components compared to 33% of urban (p-value< 0.001). This difference was predominantly driven by differences across women reporting eight or more visits.

## Discussion

In this paper, we presented a comprehensive analysis of ANC service use in Egypt over the past three decades using nationally representative data. We showed that Egypt’s improvements in ANC coverage were characterized by women’s decreasing reliance on public-sector services, an increasing number of visits during pregnancy and narrowing regional differences in coverage. Despite improvements in ANC coverage, the percentage of women who received all measured components of care in the latest survey remained very low in both sectors. In addition, we identified differences across wealth groups and regions.

The private sector provided about two-thirds of ANC at the beginning of the period under consideration, increasing to nine out of 10 women using private ANC by the most recent survey. In Egypt, a private ANC visit is estimated to cost 10 times more than a public visit [25] and public ANC services are supposed to be provided free of charge in the morning and for a low fee during the afternoon. Considering that most women have access to public health facilities [14, 26], the large private-sector market share suggests that most women consider the care delivered by public services inadequate [27] and are willing to pay additionally for services. A lack of essential medications and tests [28, 29], an unresponsive system with long waiting times, difficulties booking appointments and lack of provider choice [30] have been previously described at national level [31, 32]. These likely contribute to the increase in use of private providers [32]. In this context, private ANC use is likely to have contributed to the burden of out-of-pocket payments in the country. According to World Bank, out-of-pocket payments amounted to 62% of current health expenditure in Egypt in 2015 [33].

Meanwhile, a tenth of women did not receive any ANC in 2010–2014, particularly in Frontier Governorates and in Upper Egypt, where a large share of Egypt’s low-income households live [34]. Women in the poorest regions report distance, transportation and service fees as persistent barriers to ANC access [35]. Eligible beneficiaries may not be aware of the existence of payment exemptions to poorer families [29], while subsidies aimed at the poorest households appear to have failed to be targeted at such households [25, 36]. To support these women, it is necessary to address the existing bias in the health system towards urban areas [32], focusing the scale-up of services to the areas with the lowest coverage [31]. To ensure women’s access to services, it is fundamental to expand financial protection through social health insurance coverage for the poor and those in the informal sector [31], and ensure that mechanisms of financial support are known to users. Community health workers have been previously successful in informing communities, raising awareness of the importance of services and improving demand for them [37]. This successful experience could be replicated to inform other women of the benefits to which they are entitled.

On the other hand, women from wealthier households and women using private ANC received ever-increasing numbers of ANC visits. This phenomenon is likely driven by factors including profit-making incentives such as selling packaged ANC services with large numbers of visits and the use of unnecessary technologies [12]. Another possibility is that women prefer a higher number of visits, considering this a sign of better, more attentive care. These hypotheses would be consistent with the over-medicalization process observed in the Egyptian system, for example in the form of increased percentages of caesarean sections, [38], which reached 52% of deliveries nationally in 2014 [18].

Despite the increase in the average number of visits, less than a third of women attending public or private ANC reported receiving all assessed components of care. The percentage of women with eight or more visits receiving all such care components was also remarkably low in both the public and private sectors. This is particularly serious considering that all the components assessed should be provided during the first visit, and many of the care components should be performed multiple times over the course of pregnancy according to national guidelines [9, 39]. Thus, providers in both sectors seem to fail to deliver ANC according to national and international guidelines. The lack of some drugs and equipment in the public sector [28, 29, 32] might prevent some women from accessing blood and urine tests and more complex services, while women in the private sector might forego tests and care costed separately. However, four out of the seven measured components do not require sophisticated equipment. Moreover, information on danger signs was the least reported component, despite only requiring providers’ time with women. The reported gap in their provision of these four components suggests that providers’ lack of incentives or knowledge to meet patients’ needs may be partially responsible for inadequate ANC quality. Poor compliance with guidelines, including poor providers’ communication, has been previously reported in Menoufia and Alexandria [30]. A possible solution may be found in an incentive scheme that increased public providers’ likelihood of administering ANC components in Lower and Upper Egypt [40]. However, we observed that provision in private ANC, where salaries were higher, was also poor. Therefore, a combination of strategies targeting quality of care within both public and private sectors should be used to address this issue. Approaches might include the use of performance-based incentives in the public system, or further training and licensing systems focused on quality [31]. Moreover, structural observations of compliance with guidelines should be part of the quality supervision of health facilities [29].

In addition to suboptimal content of care, women from the wealthiest 40% of households and urban regions generally received more components in both public and private sectors compared to the poorest 40% and those in rural regions. Tetanus protection was an exception; vaccinations are provided in the public but not in private sector, with private users referred to the public system to get vaccinated. Reasons for these women getting more components might include women paying to receive more services, or better private care, but also represent differences between less and more educated women, the latter being more aware of the care they should receive. As a result, poorer, less educated and less empowered women might be receiving worse quality of care [25, 41]. To address the existing inequities in care content, interventions will have to focus attention on those reporting the fewest components of care. For instance, performance-based incentives in the public system might be targeted at services provided to less well-off women. Defining the content and price of service packages might also be used to ensure quality [31]. This could be combined with outreach initiatives in target communities [37], to educate women to be aware of the care they should receive. Moreover, improving the participation of informed women could contribute to improve services’ quality [31], for example measuring and placing importance on women’s satisfaction with the health services.

To track future improvements in ANC, there is a need to move away from simply measuring coverage to introduce indicators that support the monitoring and evaluation of quality in ANC [12]. Egypt has already taken steps in the right direction, implementing a antenatal surveillance program to monitor the quality and frequency of antenatal care visits [32]. However, this system also needs to have a focus on disadvantaged groups and regions in order to achieve equity [31]. Equity-focused models, disaggregated by key data, can contribute to identifying barriers and assessing progress in facilities’ capacity to provide services [42]. Future research should inform such equity-focused efforts to improve ANC quality. Questions may include assessing if poor-quality ANC is linked to specific types of providers, identifying the determinants behind poor compliance with guidelines in public and private ANC, and evaluating how the interventions’ described can improve quality and equity.

The limitations of this study are connected to our use of the DHS data and the type of analyses we conducted. Surveys only included women that had live births, thus information on women with poor outcomes (e.g., stillbirth, miscarriage, maternal death) is not captured in the study sample. Although no studies have been conducted that examine mothers’ recall of ANC in Egypt, results from different countries suggest the quality of the information collected by the DHS surveys in other areas and countries is acceptable [43–45]. Additionally, the long recall period (5 yrs) might have affected the ability of some women to accurately recall whether they received specific interventions. The analysis of components of care only represented an aspect of quality, but others such as timing of the care, or the patient-provider relation are not collected by the DHS. Information on whether the components of care were provided with the required frequency, or whether these components were performed correctly was not available.

## Conclusion

Egypt’s improvements in ANC coverage were characterized by women’s decreasing reliance on public services and a rise in the number of ANC visits. These changes suggest inadequate public ANC and an increase in out-of-pocket payments among women moving to the private sector hoping for better care. Despite improvements in ANC coverage, changes failed to translate into adequate ANC. Less than a third of women received, at least once during pregnancy, the seven essential ANC components measured by DHS. Furthermore, women from poorer households reported receiving fewer components than their wealthier counterparts. Policymakers need now to move rapidly to ensure that improvements in ANC coverage were not made in vain, and that they target differences in coverage and quality across groups in the country, and in both public and private sectors. It is only through the introduction of equity-focused interventions targeting ANC quality that the health of all Egyptian women can be improved. Further research needs to support this effort by assessing the determinants behind poor quality of ANC and evaluating potential interventions.

## Additional file

Additional file 1: **Table S1.** Study population. In total, complete data on ANC use and key variables of interest was available for 45,550 women across the five surveys, from which 30,956 were users of ANC. The characteristics of the study participants on each survey are shown in Supplementary Table 1. Study population, stratified by survey year and demographic and socio-economic characteristics. (DOCX 21 kb)

## Abbreviations

ANC: Antenatal care
FG: Frontier Governorates
RLE: Rural Lower Egypt
RUE: Rural Upper Egypt
ULE: Urban Lower Egypt
UUE: Urban Upper Egypt
WHO: World Health Organization

## References

[1] Campbell Oona MR, Graham Wendy J (2006). Strategies for reducing maternal mortality: getting on with what works. The Lancet.

[2] Carroli Guillermo, Villar José, Piaggio Gilda, Khan-Neelofur Dina, Gülmezoglu Metin, Mugford Miranda, Lumbiganon Pisake, Farnot Ubaldo, Bersgjø Per (2001). WHO systematic review of randomised controlled trials of routine antenatal care. The Lancet.

[3] Carroli G, Rooney C, Villar J. How effective is antenatal care in preventing maternal mortality and serious morbidity? An overview of the evidence. Paediatr Perinat Epidemiol [Internet]. 2001 Jan [cited 2018 Aug 26];15 Suppl 1:1–42. Available from: http://www.ncbi.nlm.nih.gov/pubmed/11243499. 10.1046/j.1365-3016.2001.0150s1001.x 11243499

[4] Villar J, Ba’aqeel H, Piaggio G, Lumbiganon P, Miguel Belizán J, Farnot U, et al. WHO antenatal care randomised trial for the evaluation of a new model of routine antenatal care. Lancet (London, England) [Internet]. 2001 May 19 [cited 2018 Aug 26];357(9268):1551–64. Available from: http://www.ncbi.nlm.nih.gov/pubmed/11377642. 10.1016/s0140-6736(00)04722-x 11377642

[5] McClure E.M., Goldenberg R.L., Bann C.M. (2007). Maternal mortality, stillbirth and measures of obstetric care in developing and developed countries. International Journal of Gynecology & Obstetrics.

[6] World Health Organization (WHO). Standards for maternal and neonatal care [Internet]. 2007 [cited 2018 Aug 26]. Available from: https://www.who.int/maternal_child_adolescent/documents/a91272/en/.

[7] Kuhnt Jana, Vollmer Sebastian (2017). Antenatal care services and its implications for vital and health outcomes of children: evidence from 193 surveys in 69 low-income and middle-income countries. BMJ Open.

[8] Abou-Zahr I, Lidia C, Wardlaw Tessa M. Antenatal Care in Developing Countries Promises, achievements and missed opportunities. 2003 [cited 2018 Aug 26];1–36. Available from: https://www.who.int/reproductivehealth/publications/maternal_perinatal_health/9241590947/en/.

[9] World Health Organization (WHO). WHO recommendations on antenatal care for a positive pregnancy experience [Internet]. WHO Press; 2016 [cited 2017 Dec 4]. Available from: https://www.who.int/reproductivehealth/publications/maternal_perinatal_health/anc-positive-pregnancy-experience/en/. 28079998

[10] Hodgins S, D’Agostino A (2014). The quality-coverage gap in antenatal care: toward better measurement of effective coverage. Glob Heal Sci Pract.

[11] Ng Marie, Fullman Nancy, Dieleman Joseph L., Flaxman Abraham D., Murray Christopher J. L., Lim Stephen S. (2014). Effective Coverage: A Metric for Monitoring Universal Health Coverage. PLoS Medicine.

[12] Benova Lenka, Tunçalp Özge, Moran Allisyn C, Campbell Oona Maeve Renee (2018). Not just a number: examining coverage and content of antenatal care in low-income and middle-income countries. BMJ Global Health.

[13] Arsenault Catherine, Jordan Keely, Lee Dennis, Dinsa Girmaye, Manzi Fatuma, Marchant Tanya, Kruk Margaret E (2018). Equity in antenatal care quality: an analysis of 91 national household surveys. The Lancet Global Health.

[14] Campbell O, Gipson R, Issa AH, Matta N, Deeb B El, Mohandes A El, et al. National maternal mortality ratio in Egypt halved between 1992–93 and 2000. Bull World Health Organ [Internet]. 2005 [cited 2017 15];83(6). Available from: https://www.ncbi.nlm.nih.gov/pmc/articles/PMC2626255/pdf/15976898.pdf PMC2626255 15976898

[15] World Health Organization, UNICEF, UNFPA, World Bank Group, United Nations. Trends in Maternal Mortality: 1990 to 2015: estimates by WHO, UNICEF, UNFPA: World Bank Group and the United Nations Population Division; 2015.

[16] Kassebaum NJ, Barber RM, Bhutta ZA, Dandona L, Gething PW, Hay SI, et al. Global, regional, and national levels of maternal mortality, 1990 to 2015: a systematic analysis for the Global Burden of Disease Study 2015 [Internet]. Vol. 388, The Lancet. 2016 [cited 2018 Aug 26]. Available from: https://www.thelancet.com/journals/lancet/article/PIIS0140-6736(16)31470-2/fulltext. 10.1016/S0140-6736(16)31470-2 PMC5224694 27733286

[17] Gipson Reginald, Mohandes Ayman El, Campbell Oona, Issa Adel Hakim, Matta Nahed, Mansour Esmat (2005). The Trend of Maternal Mortality in Egypt from 1992?2000: An Emphasis on Regional Differences. Maternal and Child Health Journal.

[18] Ministry of Health and Population of Egypt, El-Zanaty and Associates, ICF International. Egypt Demographic and Health Survey 2014 [Internet]. 2015 [cited 2018 Dec 3]. Available from: https://dhsprogram.com/pubs/pdf/FR302/FR302.pdf

[19] Boerma Ties, Ronsmans Carine, Melesse Dessalegn Y, Barros Aluisio J D, Barros Fernando C, Juan Liang, Moller Ann-Beth, Say Lale, Hosseinpoor Ahmad Reza, Yi Mu, de Lyra Rabello Neto Dácio, Temmerman Marleen (2018). Global epidemiology of use of and disparities in caesarean sections. The Lancet.

[20] Boerma JT, Bryce J, Kinfu Y, Axelson H, Victora CG, Bernstein S, et al. Mind the gap: equity and trends in coverage of maternal, newborn, and child health services in 54 Countdown countries. Lancet [Internet]. 2008 12 [cited 2018 Dec 3];371(9620):1259–67. Available from: https://www.ncbi.nlm.nih.gov/pubmed/18406860. 10.1016/S0140-6736(08)60560-7 18406860

[21] Powell-Jackson Timothy, Macleod David, Benova Lenka, Lynch Caroline, Campbell Oona M. R. (2014). The role of the private sector in the provision of antenatal care: a study of Demographic and Health Surveys from 46 low- and middle-income countries. Tropical Medicine & International Health.

[22] Al Rifai RH. Trend of caesarean deliveries in Egypt and its associated factors: Evidence from national surveys, 2005-2014. BMC Pregnancy Childbirth [Internet]. 2017 [cited 2018 Sep 3];17(1):417. Available from: http://www.ncbi.nlm.nih.gov/pubmed/29237410. 10.1186/s12884-017-1591-2 PMC5729511 29237410

[23] Filmer D, Pritchett LH. Estimating Wealth Effects without Expenditure Data-or Tears: An Application to Educational Enrollments in States of India. Demography [Internet]. 2001 Feb [cited 2018 Sep 3];38(1):115. Available from: http://www.jstor.org/stable/3088292?origin=crossref 10.1353/dem.2001.0003 11227840

[24] Rutstein SO, Johnson K. The DHS Wealth Index. DHS Comp Reports No 6. 2004;6:1–71.

[25] Benova L, Campbell OM, Ploubidis GB. A mediation approach to understanding socio-economic inequalities in maternal health-seeking behaviours in Egypt. BMC Health Serv Res [Internet]. 2015;15(1):1. Available from: 10.1186/s12913-014-0652-8. 10.1186/s12913-014-0652-8 PMC4307186 25603697

[26] Country Cooperation Strategy for WHO and Egypt. 2010 [cited 2017 Dec 15]; Available from: https://apps.who.int/iris/handle/10665/113237.

[27] Benova L, Campbell OM, Sholkamy H, Ploubidis GB. Socio-economic factors associated with maternal health-seeking behaviours among women from poor households in rural Egypt. Int J Equity Health [Internet]. 2014;13(1):111. Available from: 10.1186/s12939-014-0111-5 10.1186/s12939-014-0111-5 PMC4247707 25424200

[28] Gadallah M, Zaki B, Rady M, Anwer W, Sallam I (2003). Patient satisfaction with primary health care services in two districts in lower and upper Egypt. EMHJ - East Mediterr Heal J [Internet].

[29] World Bank. Management and service quality in primary health care facilities in the Alexandria and Menoufia governorates [Internet]. 2010 [cited 2017 Dec 8]. Available from: http://documents.worldbank.org/curated/en/814731468021569255/pdf/693450ESW0P112000June025020100lerg.pdf

[30] Zaky HHM, Khattab HAS, Ma NN. Experiences of women using reproductive health services in Egypt: One health system in two governorates. Qual Prim Care [Internet]. 2010 [cited 2017 Mar 20];18(5):345–52. Available from: https://www.ncbi.nlm.nih.gov/pubmed/21114915. 21114915

[31] World Bank. A Roadmap to Achieve Social Justice in Health Care in Egypt [Internet]. 2015 [cited 2017 Dec 8]. Available from: https://www.worldbank.org/content/dam/Worldbank/Feature%20Story/mena/Egypt/Egypt-Doc/egy-roadmap-sj-health.pdf.

[32] UNFPA. Population Situation Analysis of Egypt [Internet]. 2016 [cited 2018 Sep 3]. Available from: https://egypt.unfpa.org/en/publications/population-situation-analysis-egypt-2016-report.

[33] World Bank Group. World Development Indicators | DataBank [Internet]. 2018 [cited 2018 Dec 21]. Available from: http://databank.worldbank.org/data/reports.aspx?source=world-development-indicators.

[34] Ghanem H. Improving regional and rural development for inclusive growth in Egypt [Internet]. Vol. 67, Global Economy and Development at Brookings. 2015. Available from: https://www.brookings.edu/wp-content/uploads/2016/06/Arab-EconPaper2Hafez-FINAL.pdf.

[35] Chiang C, Labeeb SA, Higuchi M, Mohamed AG, Aoyama A. Barriers to the use of basic health services among women in rural southern Egypt (Upper Egypt). Nagoya J Med Sci [Internet]. 2013 [cited 2017 Dec 8];75(3–4):225–31. Available from: http://www.ncbi.nlm.nih.gov/pubmed/24640178. PMC4345669 24640178

[36] Rashad A, Sharaf M (2015). Who benefits from public healthcare subsidies in Egypt?. Soc Sci [Internet].

[37] Brasington Angela, Abdelmegeid Ali, Dwivedi Vikas, Kols Adrienne, Kim Young-Mi, Khadka Neena, Rawlins Barbara, Gibson Anita (2016). Promoting Healthy Behaviors among Egyptian Mothers: A Quasi-Experimental Study of a Health Communication Package Delivered by Community Organizations. PLOS ONE.

[38] Khadr Z. Monitoring socioeconomic inequity in maternal health indicators in Egypt: 1995-2005. Int J Equity Health [Internet]. 2009 Nov 8 [cited 2017 Dec 15];8:38. Available from: http://www.ncbi.nlm.nih.gov/pubmed/19895706. 10.1186/1475-9276-8-38 PMC2781806 19895706

[39] Egyptian guidelines.

[40] Huntington D, Zaky HHM, Shawky S, Fattah FA, El-Hadary E. Impact of a service provider incentive payment scheme on quality of reproductive and child-health services in Egypt. J Health Popul Nutr [Internet]. 2010 [cited 2017 Dec 4];28(3):273–80. Available from: http://www.ncbi.nlm.nih.gov/pubmed/20635638. 10.3329/jhpn.v28i3.5556 PMC2980892 20635638

[41] Zaky HHM, Armanious DM, Hussein MA. Testing for the endogenous nature between women’s empowerment and antenatal health care utilization: evidence from a cross-sectional study in Egypt. Biomed Res Int [Internet]. 2014 [cited 2017 Dec 4];2014:403402. Available from: http://www.ncbi.nlm.nih.gov/pubmed/25140310. 10.1155/2014/403402 PMC4129960 25140310

[42] UNICEF. Reaching Universal Health Coverage through District Health System Strengthening: Using a modified Tanahashi model sub-nationally to attain equitable and effective coverage [Internet]. Maternal, Newborn and Child Health Working Paper, UNICEF Health Section. 2013 [cited 2017 Dec 8]. Available from: https://www.unicef.org/health/files/DHSS_to_reach_UHC_121013.pdf.

[43] Liu Li, Li Mengying, Yang Li, Ju Lirong, Tan Biqin, Walker Neff, Bryce Jennifer, Campbell Harry, Black Robert E., Guo Yan (2013). Measuring Coverage in MNCH: A Validation Study Linking Population Survey Derived Coverage to Maternal, Newborn, and Child Health Care Records in Rural China. PLoS ONE.

[44] Tomeo Catherine A., Rich-Edwards Janet W., Michels Karin B., Berkey Catherine S., Hunter David J., Frazier A. Lindsay, Willett Walter C., Buka Stephen L. (1999). Reproducibility and Validity of Maternal Recall of Pregnancy-Related Events. Epidemiology.

[45] McCarthy KJ, Blanc AK, Warren CE, Kimani J, Mdawida B, Ndwidga C. Can surveys of women accurately track indicators of maternal and newborn care? A validity and reliability study in Kenya. J Glob Health [Internet]. 2016 [cited 2017 Dec 15];6(2):020502. Available from: http://www.ncbi.nlm.nih.gov/pubmed/27606061. 10.7189/jogh.06.020502 PMC5012235 27606061

