# Supplementary material for: Temporal and regional variations in use, equity and quality of antenatal care in Egypt: a repeat cross-sectional analysis using Demographic and Health Surveys
Source: BMC Pregnancy Childbirth. 2019 Jul 26;19:268. doi: 10.1186/s12884-019-2409-1 (PMC6660959; doi:10.1186/s12884-019-2409-1)
Supplement: Supplementary file 1 — Table S1. Study population. In total, complete data on ANC use and key variables of interest was available for 45,550 women across the five surveys, from which 30,956 were users of ANC. The characteristics of the study participants on each survey are shown in Supplementary Table 1. Study population, stratified by survey year and demographic and socio-economic characteristics. (DOCX 21 kb) [file 12884_2019_2409_MOESM1_ESM.docx]

|  | **1991-1995** | | **1996-2000** | | **2001-2005** | | **2004-2008** | | **2010-2014** | | **Total** | |
| --- | --- | --- | --- | --- | --- | --- | --- | --- | --- | --- | --- | --- |
|  | **Weighed %** | **N** | **Weighed %** | **N** | **Weighed %** | **N** | **Weighed %** | **N** | **Weighed %** | **N** | **Weighed %** | **N** |
| **In need of ANC care** | 100% | 7,992 | 100% | 7,996 | 100% | 9,985 | 100% | 8,030 | 100% | 11,493 | 100% | 45,496 |
| **Age** |  |  |  |  |  |  |  |  |  |  |  |  |
| 15-19 | 4% | 336 | 4% | 285 | 4% | 391 | 3% | 275 | 3% | 322 | 4% | 1,609 |
| 20-29 | 50% | 3947 | 52% | 4094 | 55% | 5420 | 58% | 4571 | 55% | 6233 | 54% | 24,265 |
| 30-39 | 37% | 2992 | 37% | 3016 | 35% | 3493 | 33% | 2685 | 37% | 4316 | 36% | 16,502 |
| 40-49 | 9% | 717 | 7% | 601 | 7% | 681 | 6% | 499 | 5% | 622 | 7% | 3,120 |
| **Education** |  |  |  |  |  |  |  |  |  |  |  |  |
| No education | 44% | 3,705 | 39% | 3,082 | 30% | 3,208 | 25% | 2,185 | 18% | 1,930 | 30% | 14,110 |
| Some primary | 22% | 1,655 | 16% | 1,232 | 13% | 1,267 | 10% | 821 | 9% | 982 | 13% | 5,957 |
| Primary complete / some secondary | 28% | 2,217 | 37% | 3,009 | 46% | 4,560 | 51% | 4,052 | 57% | 6,662 | 45% | 20,500 |
| Secondary complete / higher | 6% | 415 | 9% | 673 | 11% | 950 | 13% | 972 | 16% | 1,919 | 11% | 4,929 |
| **Marital status** |  |  |  |  |  |  |  |  |  |  |  |  |
| Married | 98% | 7,834 | 98% | 7,856 | 98% | 9,779 | 98% | 7,888 | 98% | 11,279 | 98% | 44,636 |
| Separated, Divorced, Widowed or not living together | 2% | 158 | 2% | 140 | 2% | 206 | 2% | 142 | 2% | 214 | 2% | 860 |
| **Type of residence** |  |  |  |  |  |  |  |  |  |  |  |  |
| Rural | 59% | 4992 | 60% | 4605 | 62% | 6174 | 62% | 5012 | 68% | 6724 | 63% | 27507 |
| Urban | 41% | 3000 | 40% | 3391 | 38% | 3811 | 38% | 3018 | 32% | 4769 | 37% | 17989 |
| **Household wealth** |  |  |  |  |  |  |  |  |  |  |  |  |
| 1 | 22% | 2008 | 20% | 1599 | 20% | 2327 | 19% | 1766 | 17% | 2065 | 19% | 9765 |
| 2 | 20% | 1636 | 19% | 1449 | 20% | 2094 | 20% | 1640 | 19% | 2109 | 20% | 8928 |
| 3 | 21% | 1570 | 20% | 1634 | 21% | 1973 | 21% | 1667 | 25% | 2346 | 22% | 9190 |
| 4 | 19% | 1413 | 22% | 1714 | 21% | 1915 | 21% | 1515 | 21% | 2431 | 21% | 8988 |
| 5 | 19% | 1365 | 19% | 1600 | 18% | 1676 | 19% | 1442 | 17% | 2542 | 18% | 8625 |
| **Region of residence** |  |  |  |  |  |  |  |  |  |  |  |  |
| Urban governorates | 19% | 1131 | 17% | 1381 | 15% | 1545 | 16% | 1074 | 11% | 1735 | 15% | 6866 |
| Urban Lower Egypt | 10% | 644 | 12% | 858 | 10% | 708 | 10% | 711 | 9% | 1174 | 10% | 4095 |
| Rural Lower Egypt | 29% | 1666 | 31% | 2150 | 31% | 2165 | 34% | 2280 | 39% | 3138 | 33% | 11399 |
| Urban Upper Egypt | 11% | 828 | 11% | 826 | 13% | 1268 | 11% | 945 | 11% | 1382 | 11% | 5249 |
| Rural Upper Egypt | 29% | 3027 | 28% | 2241 | 30% | 3809 | 27% | 2562 | 29% | 3331 | 29% | 14970 |
| Frontier Governorates | 1% | 696 | 1% | 540 | 1% | 490 | 1% | 458 | 1% | 733 | 1% | 2917 |
|  |  |  |  |  |  |  |  |  |  |  |  |  |
